# Presence of fruits decreases probability of retaining flowers in a sequentially flowering plant

**DOI:** 10.1093/aobpla/ply033

**Published:** 2018-05-23

**Authors:** Shivani Jadeja, Brigitte Tenhumberg

**Affiliations:** School of Biological Sciences, University of Nebraska, Lincoln, NE, USA

**Keywords:** Architectural effects hypothesis, flower retention, fruit abortion, life-history strategy, resource allocation, sink strength hypothesis, yucca, *Yucca glauca*

## Abstract

Both intrinsic and extrinsic plant processes affect the fate of flowers along an inflorescence in sequentially flowering plants. We investigated whether the intrinsic process of competition for limited resource between fruits and flowers owing to resource preemption or sink strength of basal fruits, or architectural effects due to positional differences in the probability of retaining flowers, explains a lower probability of retaining distal flowers in *Yucca glauca*. Further, we investigated how the extrinsic process of seed herbivory interacts with the plant’s intrinsic processes of flower retention. We carried out a field experiment to compare flower retention among nine combinations of three inflorescence treatments (basal flowers only, distal flowers only, distal flowers with presence of basal fruits) and three ovule damage treatments (no, low and high) that serve as a cue for potential future seed herbivory. Also, we quantified flower retention in naturally pollinated inflorescences. Experimental results showed that the probabilities of retaining basal and distal flowers in the absence of basal fruits were similar, thus rejecting the architectural effects hypothesis. Further, in the presence of basal fruits that were in their initial stages of growth, the probability of retaining distal flowers decreased, which supports the sink strength hypothesis. We did not see an effect of ovule damage. In naturally pollinated inflorescences, the probability of retaining distal flowers decreased with increasing number of basal fruits. Results suggest that basal fruits constitute strong resource sinks reducing the probability of retaining distal flowers. Previous studies have tested this mechanism in cultivated plants. Our study shows evidence for this mechanism in a wild flower population.

## Teaser

We investigated mechanisms underlying variation in fruiting patterns of sequentially flowering plants using the wild flower *Yucca glauca* (Family: Agavaceae). These plants start opening flowers on the bottom of an inflorescence and then keep opening new flowers all the way to the top. Our results show that plants are more likely to abort distal flowers when growing basal fruits are present. This is likely because limited fruiting resources are disproportionately accumulated by basal fruits that are stronger resources sinks than flowers.

## Introduction

Both intrinsic and extrinsic plant processes affect the fate of flowers along an inflorescence in sequentially flowering plants (Lloyd 1980; Stephenson 1981; Diggle 1995). Intrinsic processes relate to a plant’s physiology and the position of flowers along an inflorescence and extrinsic plant processes relate to environmental variables including resource and pollen availability, and herbivory. Widely tested hypotheses for intrinsic plant processes that affect the fate of flowers are related to competition for limited resources—resource preemption hypothesis and sink strength hypothesis (Stephenson 1981; Lee 1988). Developing fruits from early-opening basal flowers have a temporal advantage over distal flowers and may preempt resources that may be available for distal flowers (resource preemption hypothesis). In plants where distal flowers open while basal fruits are still growing, basal fruits that have become strong resource sinks deprive distal flowers of resources. Consequently, plants abort distal flowers in the presence of basal fruits, which has been observed in many sequentially flowering plants, including *Prunus mahaleb* (Guitián 1994) and *Phaseolus vulgaris* (Tamas *et al.* 1979). As a result, inflorescences produce more fruits from basal flowers.

Selective development of fruits on basal positions on an inflorescence may also be explained by the architectural effects hypothesis, according to which flowers at distal positions have an inherently lower probability of developing into fruits owing to quantitative architectural or positional differences along the inflorescence, independent of fruits developed from basal flowers (Diggle 1995). Shift in the probability of maturing a fruit along an inflorescence owing to architectural effects has been shown in *Narthecium asiaticum* (Ishii and Sakai 2002) and *Myrosmodes cochleare* (Berry and Calvo 1991). The proximate mechanisms of architectural effects are not yet known, but may involve decreasing quantity of vascular tissue from basal to distal flower positions or differences in the size of floral organs along the inflorescence (Diggle 2014).

We tested whether resource competition related sink strength/resource preemption, and/or flower position related architectural effects hypotheses explain the probability of basal and distal flowers developing into fruits in sequentially flowering wild plants using *Yucca glauca* (soapweed yucca, Family: Agavaceae). The majority of studies testing these hypotheses used cultivated plants (for examples see reviews by Stephenson 1981 and Diggle 2003). Evidence shows wild plants experience different environments compared to cultivated plants resulting in drastic differences in their population dynamics (Eager *et al.* 2013). Our study uses a wild plant that has been extensively studied mainly to understand its specialized pollination mutualism (Pellmyr 2003). For brevity, we refer to flowers developing into mature fruits as flower retention following previous studies (Humphries and Addicott 2000, 2004).

We only know of a single study that has tested the architectural effects hypothesis in a *Yucca* sp. (Huth and Pellmyr 1997). In this study, researchers manipulated *Yucca filamentosa* inflorescences to obtain only early- and only late-opening flowers by removing all other flowers and compared flower retention with unmanipulated inflorescences. All flowers were hand-pollinated to remove the effect of pollen limitation. Manipulated inflorescences retained a similar proportion of flowers to unmanipulated inflorescences (Huth and Pellmyr 1997). While both sink strength and architectural differences may influence flower retention in the same plant, Huth and Pellmyr’s (1997) experiment suggests that the probability of *Y. filamentosa* retaining fruits is plastic along an inflorescence, and unlikely due to architectural differences. In this study, we tested the generality of these results with a different *Yucca* species. Flower retention in *Y. glauca* might differ from *Y. filamentosa* because *Y. filamentosa* inflorescences are branched and as a result individual plants produce a much larger number of flowers.

Intrinsic plant processes may interact with extrinsic processes such as herbivory, pollen limitation, nutrient availability and weather. Major herbivores of *Yucca* spp. are their obligate nursery pollinators (Riley 1892; Pellmyr 2003). Nursery pollinators are pollinators that also lay eggs in the flowers they pollinate, and their larvae feed on the produced seeds. While the survival of nursery pollinator larvae is essential for host plants to produce fruits in future flowering seasons, plants may not benefit from producing fruits with a high number of pollinator larvae. It is well established that *Yucca* spp. host plants selectively abort flowers with a high number of pollinator eggs and selectively retain fruits with the potential of producing a high proportion of intact viable seeds (Pellmyr and Huth 1994; Humphries and Addicott 2000; Pellmyr 2003; Shapiro and Addicott 2004). Pollinator offspring cannot develop in aborted flowers.

To reduce the loss of tissue to herbivory, plants are known to respond to potential herbivory using early cues such as ovipositions by herbivores by aborting infected organs or activating chemical defences (Pashalidou *et al.* 2015). The number of ovipositions in a flower is indicative of the expected number of seed-consuming larvae a fruit will contain. In *Yucca* spp., with each oviposition event the pollinator damages (punctures) some ovules. Host plants use the extent of this ovule damage as a cue for the expected level of herbivory and may abort flowers before investing a significant amount of resources in developing fruits (Fuller 1990; Marr and Pellmyr 2003). The goal of this manuscript is to use a combination of field experiments and observations to unravel the causes underlying lower flower retention and its interaction with ovule damage.

We evaluated the following hypothesis: ovule damage from ovipositions causes chemical changes, which may trigger host plants to abort those flowers. As a consequence, flowers with a high number of ovipositions have a consistently low probability of retention, irrespective of the plant’s intrinsic process of flower retention. Alternatively, it is possible that the number of eggs per flower interacts with the plant’s intrinsic processes, i.e. basal and distal flowers may differ in their response to a high number of ovipositions. This would be consistent with earlier conjectures stating that ovule damage may reduce the resource sink strength of flowers (Marr and Pellmyr 2003; Shapiro and Addicott 2004). If sink strength determines retention of flowers with few ovipositions, we expect both basal flowers and distal flowers without basal fruits to have a high probability of flower retention. However, in the presence of basal fruits, we expect distal flowers with few ovipositions to show a low probability of retention. In contrast, if architectural effects determine flower retention, we expect basal flowers with few ovipositions to have a higher probability of retention, and distal flowers to have a low probability of retention, independent of the presence of basal fruits. Further, when flowers receive high ovipositions, we expect a decrease in these probabilities of retaining flowers. So, high and low probabilities of retaining flowers when ovipositions are few will decrease to medium and very low probabilities of retaining flowers, respectively, when ovipositions are many.

Flower retention in the field is more complicated because of possible pollen limitation, and uncertainty in herbivory over the flowering season. In our field experiment, we controlled for these factors by hand-pollinating flowers and protecting inflorescences from herbivory, respectively. To gain insights into flower retention in naturally pollinated inflorescences, we studied how the host plant’s intrinsic processes work in tandem with extrinsic processes to affect flower retention along the inflorescence. In contrast to our experiment, naturally pollinated inflorescences likely varied in pollen availability and the number of basal fruits that could divert resources from distal flowers. We predicted that, if sink strength of basal fruits decreases the chances of retaining distal flowers, the probability of retaining naturally pollinated distal flowers will decrease with increase in the number of basal fruits. We could not use the naturally pollinated inflorescences to test the architectural effects hypothesis because it is not possible to tease apart whether the differences in retention of basal and distal flowers are owing to architectural effects, sink strength of basal fruits, pollen limitation and/or herbivory. Hence, we relied solely on our experiment to test the architectural effects hypothesis.

## Methods

### Study system

We used the *Y. glauca* and its obligate pollinator *Tegeticula yuccasella* (yucca moth) as our study system. Both species are unprotected and abundant in their habitat. *Yucca* spp. and *Tegeticula* spp. are native to arid habitats across North and South America. *Yucca* spp. propagate via seeds and vegetatively through ramets and lateral buds that give rise to new rosettes that are genetic clones of the parent plants. Clones can remain connected underground through rhizomes and may share resources. Typically, each rosette grows for multiple years before it is capable of sexual reproduction (Kingsolver 1986). For sexual reproduction, a *Yucca* spp. rosette gives rise to an inflorescence during the summer. The inflorescence of *Y. glauca* is a raceme that may produce 15–240 buds (Kingsolver 1986; Svensson *et al.* 2011; S. Jadeja, pers. obs.). The *Y. glauca* flowering period is usually 15–30 days long during which flowers open from the bottom of the raceme to the top (Kingsolver 1984). After flowering, the rosette dies (Kingsolver 1986). Old rosettes are replaced by one or more new rosettes, allowing yucca clumps to expand and persist for years.

*Yucca* spp. flowers are most receptive for 1–2 days after opening during which a female *Tegeticula* sp. may lay eggs in the flower’s ovary and actively pollinate it (Dodd and Linhart 1994; Huth and Pellmyr 1997; Humphries and Addicott 2000). Within 7–10 days after oviposition, pollinator eggs hatch and feed on the developing seeds within the maturing host plant ovary (Huth and Pellmyr 1999). The number of ovules damaged increases with increasing number of *Tegeticula* spp. ovipositions (Marr and Pellmyr 2003; Shapiro and Addicott 2003). Ovules are not only damaged by female *Tegeticula* spp. during oviposition but also by *Carpophilus* sp. (florivorous beetles) and their larvae (Huth and Pellmyr 1997). The damage to ovules serves as a cue for flower abortion (Marr and Pellmyr 2003). Ninety-five percent of the flowers that the plant aborts are aborted within 7 days after they open (Pellmyr and Huth 1994). Thereafter, plants rarely abort fruits even if they carry a large number of seed herbivorous larvae. On average, *Yucca* spp. set fruit from <15 % of their flowers (Udovic and Aker 1981; Pellmyr 1997; Addicott 1998), primarily due to limited resources (Huth and Pellmyr 1997). Excess flower production along with abortion of fruits has been shown to be an evolutionarily stable strategy in nursery pollinator mutualisms like yucca-yucca moth mutualisms where pollinators also consume seeds (Holland *et al.* 2004).

### Hand-pollination

We eliminated pollen limitation by hand-pollinating all experimental plants. To obtain donor flowers for hand-pollination, we protected donor *Y. glauca* inflorescences using a mesh sleeve made of fine tulle fabric that prevented pollen collection by *T. yuccasella* and reduced damage by *Carpophilus* sp. We collected fresh donor flowers, usually the topmost herbivore-free flowers on an inflorescence, from donor plants at least 25 m away from the recipient plant (except one recipient for which donor flowers were collected from 10 m away). We placed collected flowers away from direct sunlight in a plastic container lined with paper towels. We utilized pollen from donor flowers within 3 h of collection. *Yucca glauca* pollen is known to be viable for 4 days (Dodd and Linhart 1994).

To hand-pollinate flowers, we used a toothpick to collect pollen from one anther lobe of a donor flower and placed it on the stigmatic opening of the recipient flower. One anther lobe produces a few thousand pollen grains (S. Jadeja, pers. obs.) which is more than sufficient pollen to pollinate all ovules within a single *Y. glauca* ovary that contains on average nearly 300 ovules (Addicott 1986). Next, we used a size 0 brush to push the pollen well inside the stylar canal. We pollinated all experimental flowers within an inflorescence with pollen from the same pollen donor to control for the effect of differences in pollen quality on flower retention. Further, we thoroughly cleaned both the toothpick and the brush between pollen donors to prevent the transfer of mixed pollen genotypes.

### Artificial oviposition

We used artificial wounding to mimic different levels of ovipositions following the method described by Marr and Pellmyr (2003). The level of oviposition is an early indicator of the extent of potential seed herbivory a fruit may experience when oviposited eggs hatch. We constructed an artificial ovipositor by attaching a microneedle (minutien insect pin) to a matchstick, as done by Marr and Pellmyr (2003). The artificial ovipositor’s thickness is similar to that of the *T. yuccasella* ovipositor (Marr and Pellmyr 2003). We quantified the thickness of the ovary wall from the groove within the ovary along the middle of the ovary for 55 flowers (2–5 flowers from 19 inflorescences) from the study site. This is where we have observed *T. yuccasella* inserting their ovipositor. The thickness of the ovary wall was 1.96 ± 0.04 mm (mean ± SE) based on which we constructed 2.5-mm-long artificial ovipositors such that they were long enough to damage ovules.

Following Marr and Pellmyr (2003), we applied 0, 6 or 24 artificial ovipositions to mimic no, low and high number of ovipositions, respectively, to each experimental flower on an inflorescence. Twenty-four ovipositions is close to the maximum of 30 ovipositions by a single female observed once at this study site in 2014 (S. Jadeja, pers. obs.). We applied the artificial oviposition treatment at the groove in the middle of the ovary as done by the natural pollinator. We distributed all artificial ovipositions equally across the six compartments of the ovary (locules) that are clearly differentiated by the anther filaments.

### Flower manipulation experiment

We carried out a field experiment from early May to early July 2015 at the Cedar Point Biological Station (CPBS), Keith County, NE, USA. We selected 114 undamaged *Y. glauca* inflorescences that had yet to begin flowering and protected them from deer herbivory using tomato cages with sides wrapped with 2.54 cm hex netting. It was not possible to select inflorescences of similar size or flowering period for this study owing to the difficulty in finding sufficient number of inflorescences free from herbivory by deer and florivorous beetles. Among inflorescences from visibly identifiable clones, we haphazardly selected only one focal inflorescence for our experiment. In addition, we removed buds from the remaining clonal inflorescences to minimize fruit abortion on focal inflorescences due to division of resources among clonal inflorescences.

To prevent *T. yuccasella* from visiting flowers on the selected inflorescences, we covered inflorescences with mesh sleeves made of fine tulle fabric. If an inflorescence had already opened a few early flowers, we broke the flowers off before placing the sleeve. We also removed all visible florivorous beetles (*Carpophilus* sp.) from the inflorescence.

It was not possible to remove all florivorous beetles from all protected inflorescences, and some excluded florivorous beetles were able to damage flowers that abutted the mesh sleeve. Since *Yucca* spp. abort flowers damaged by florivorous beetles (Huth and Pellmyr 1997), we discarded visibly beetle-infested and damaged inflorescences from the experiment. Overall, we discarded 30 inflorescences, leaving 84 inflorescences for the experiment. All flowers used in the experiment were less than two nights old, after which they may no longer be receptive.

We randomly assigned inflorescences to one of three treatments of flower position and presence of fruits, (i) early-opening flowers with buds above, (ii) late-opening flowers without existing fruits on the inflorescence and (iii) late-opening flowers with basal fruits on the inflorescence **[see****Supporting Information—Fig. S1****]**. Inflorescences assigned to each treatment were statistically similar in both the size of their rosettes (basal diameter in mm, pairwise comparisons using Wilcoxon rank tests, *P* > 0.5) and the number of buds (pairwise comparisons using Wilcoxon rank tests, *P* > 0.3). On average, the experimental inflorescences were on rosettes with 63 ± 1 mm (mean ± SE) basal diameter and produced 49 ± 2 buds. The flowering period of these inflorescences was ~15 days (S. Jadeja, pers. obs.). We were unable to quantitatively determine the flowering period of experimental inflorescences because the length of the flowering period may have changed as a result of our manipulations that included flower and bud removal.

We manipulated inflorescences to obtain their assigned treatments (henceforth, inflorescence treatments). For the inflorescence treatment—late-opening flowers with already-existing fruits, we used one to three basal fruits formed by hand-pollinating flowers. Out of the 18 inflorescences with basal fruits, eight inflorescences had three fruits, five inflorescences had two fruits and the remaining five inflorescences had one fruit. When experimental flowers were receptive, basal fruits were only slightly larger than the flower’s ovary and were still at an early stage of growth, which is necessary to test whether the strong sink strength of developing basal fruits influences retention of distal flowers. On each manipulated inflorescence, we used three experimental flowers that we hand-pollinated. On inflorescences with basal fruits the time lag between hand-pollinating basal flowers to obtain basal fruits and distal experimental flowers was 6 ± 0.3 days (mean ± SE). The small number of flowers and fruits used in the experiment reduced the chance of flower abortion due to limited resources. The maximum number of fruits that could be produced on a focal inflorescence was four to six fruits (three fruits developing from experimental flowers + one to three already-existing fruits), which equals the average number of fruits produced on an inflorescence at our study site (see results for fruit production of naturally pollinated inflorescences). Further, naturally pollinated inflorescences on rosettes with sizes similar to rosettes used in the experiment produced up to 18 fruits.

We randomly assigned each manipulated inflorescence to an oviposition treatment—no, low or high oviposition. This yielded nine treatments, which we distributed as evenly as possible across early- and late-flowering inflorescences. Discarding inflorescences with beetle damage and inflorescences that did not form basal fruits required for the treatment with prior fruiting resulted in unequal sample sizes among treatments **[see****Supporting Information—Fig. S1****]**.

Ten days after applying the oviposition treatment and hand-pollinating experimental flowers, we recorded the number of retained flowers. Since 95 % of flower abortions in *Y. filamentosa* take place within 7 days (Pellmyr and Huth 1994) and Dodd and Linhart (1994) recorded *Y. glauca* fruit set 7–10 days after pollination, we considered any fruit remaining after 10 days as retained flowers. We collected fruits developed from experimental flowers 25 days after hand-pollination. We weighed fruits immediately after collection to determine whether our treatments affected fruit mass.

We used collected fruits to check whether our artificial oviposition treatment damaged ovules as intended. Ovules damaged during oviposition are white and infertile, whereas fertile ovules are black. We quantified the number of infertile white seeds from a haphazardly selected subset of collected fruits. Fruits were from inflorescences without basal fruits, and with no and high artificial oviposition treatments (*n* = 10 and 12, respectively). We expected the artificial oviposition treatment to increase the number of white seeds.

### Flower retention and fruit size in natural population

We used naturally pollinated *Y. glauca* inflorescences to determine the effect of the number of already-initiated basal fruits on the probability of flower retention. At the end of the *Y. glauca* flowering season in July 2015, we sampled a 55 × 25 m patch of *Y. glauca* on the northeast slope of the Kingsley dam at Lake McConaughy, Keith County, NE. The patch consisted of 106 visibly distinct *Y. glauca* clumps representing one or more clonal rosettes. This patch is 5 km from CPBS where we carried out the field experiment. The patch had 90 inflorescences of which 15 inflorescences were either damaged or used for another study. Of the remaining 75 inflorescences, we only used inflorescences with at least one fruit (57 inflorescences) for our analyses.

We measured the basal diameter of the rosette of each inflorescence which is likely an indicator the resources available to the inflorescence. Next, we quantified the number of buds produced on each inflorescence by counting the number of persistent pedicels (remnant flower stalks) and fruits. For each inflorescence, we recorded the position of each flower and whether the flower was retained. For example, we gave the fruits formed from the 1st and 10th flowers from the bottom of the inflorescence positions 1 and 10, respectively. In some cases, one axil could produce two fruits in which case we haphazardly gave fruits consecutive position values. In addition, for each distal flower, i.e. each flower from the top third flowers of the inflorescence, we recorded the number of fruits formed at flower positions below it (basal fruits).

To gain insights into variation in resource allocation to fruits along an inflorescence, we quantified the size of fruits. Out of the 57 fruiting inflorescences at the *Y. glauca* patch, we collected fruits from 30 inflorescences in late July 2015. Collected fruits were from inflorescences distributed across the patch and across the range of the total number of fruits produced by inflorescences. We labelled each collected fruit with the identity of the inflorescence and flower position, and transported them to our laboratory at the University of Nebraska–Lincoln. We allowed fruits to air dry at room temperature. Approximately 4 months after the fruits were collected we determined indices of their size—fruit mass and length, which in turn are indices of the plant’s resource allocation to the retained flower. We weighed the fruits, and measured their length from the base to the tip of the remnant style. The mass of fruits was strongly correlated with its length (*r* = 0.78, *n* = 229, *P* < 0.0001). Since fruit mass decreases with increasing seed consumption by pollinator larvae and non-pollinating seed predators, fruit length is a more reliable indicator of the plant’s investment in seed production than fruit mass. Hence, we used fruit length as a proxy for the fruit size in our analysis. Further, we recorded the following indices of pollinator oviposition and pollination in the collected fruits, which likely influence fruit size: (i) The number of locules with constrictions (out of six locules) on each fruit, which occur when many ovules are damaged at the site of pollinator oviposition (Riley 1892; Shapiro and Addicott 2003). (ii) The number of fruits tapering or rounded at the base indicating that ovules were not fertilized most likely due to insufficient pollen grains (Humphries and Addicott 2000).

### Statistical analysis

#### Flower manipulation experiment

We analysed the probability of flower retention from our experimental data using a generalized linear mixed-effects model (GLMM) with binomial errors. The proportion of flowers retained (number of fruits retained out of the three experimental flowers), which follows a binomial distribution, was the response variable. Inflorescence identity was an observation-level random effect that accounted for overdispersion (Harrison 2015). Predictor variables were inflorescence treatment (early flowers with buds above, late flowers with no fruits and late flowers with basal fruits), level of artificial ovipositions (no, low and high) and their interaction. We analysed the average mass of fruits from experimental flowers that retained at least one flower using a linear model (LM) with the same predictor variables as those for fruit retention. We used backward model selection to eliminate predictors that did not significantly influence flower retention and fruit mass, respectively. Further, we carried out *post hoc* analysis of all categorical variables in the final model using Tukey’s pairwise comparisons.

To determine whether our artificial oviposition treatment was effective, we used a generalized linear model (GLM), with quasipoisson family of errors to account for overdispersion. The number of white seeds was the response variable and oviposition treatment (high or no level of oviposition) was the predictor variable.

#### Flower retention and fruit size in natural population

Similar to our field experiment, we expected the probability of retaining distal flowers to decrease with increasing number of basal fruits in naturally pollinated inflorescences. To test our expectation, we constructed a GLMM with binomial error distribution where the response variable was whether or not a top flower was retained, and the predictor variables were the number of basal fruits and the basal diameter of the inflorescence’s rosette (an index of rosette size). Each inflorescence had multiple top flowers. We accounted for the repeated measures by using inflorescence identity as a random effect.

We examined the reproductive performance of inflorescences in relation to the size of rosettes, which is an indicator of resources available to the inflorescences. We constructed two GLMs with Poisson error distributions. The response variable for one was the number of buds and for the other was the number of fruits, and the predictor variable in both was the basal diameter of the inflorescence’s rosette.

Fruit length is an index of fruit size and the resources plants allocate to fruits. We analysed predictors of fruit length from the naturally pollinated *Y. glauca* inflorescences using a linear mixed-effects model (LMM) where the random effect was inflorescence identity to account for repeated measures within inflorescences. The predictor variables were basal diameter of the inflorescence’s rosette, the position of the fruit (bottom, middle or top), whether the fruit had a tapering base, and the number of locules with constrictions.

We carried out all statistical analyses in R version 3.4.2 (R Core Team 2017), using R packages lme4 (Bates *et al.* 2015) and nlme (Pinheiro *et al.* 2016). The data are available from the figshare data repository: https://doi.org/10.6084/m9.figshare.c.3746510.v2 (Jadeja and Tenhumberg 2018).

## Results

### Flower manipulation experiment

Overall, 45.6 % of the 252 hand-pollinated experimental flowers were retained during the experiment. Across inflorescences, nearly 30 % of the inflorescences did not retain any flowers while nearly 23 % of the inflorescences retained all three experimental flowers.

**Figure 1.**
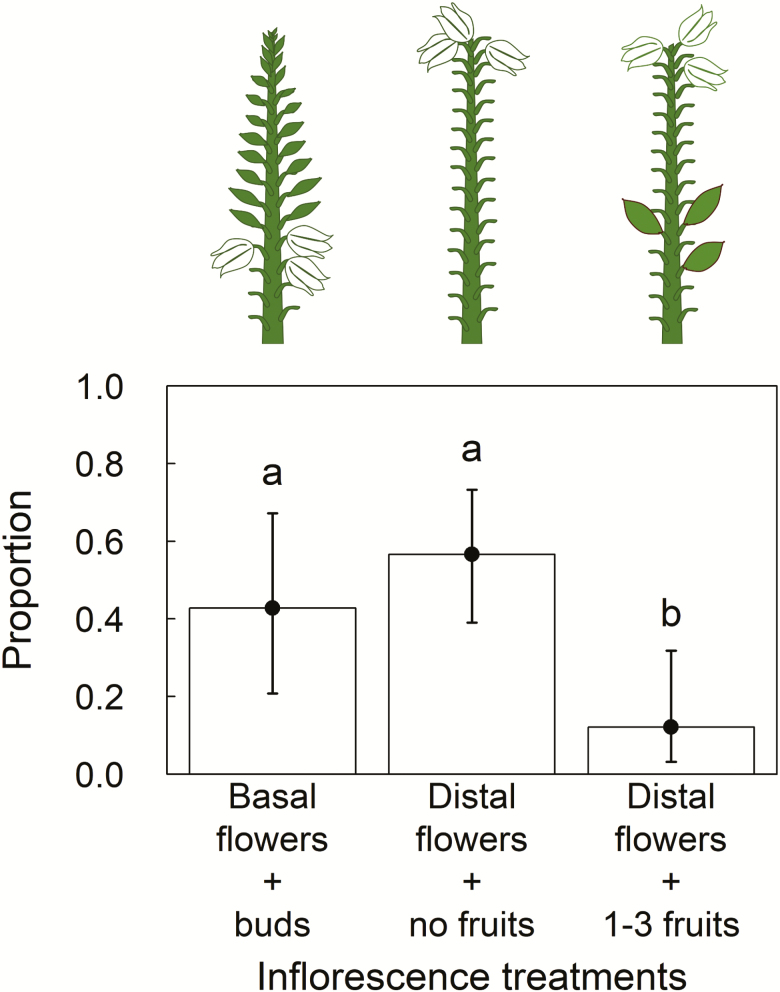
The proportion of experimental flowers retained out of three flowers under each inflorescence treatment. Inflorescence treatments (basal flowers with buds above, distal flowers with no fruits and distal flowers with already-existing basal fruits) are shown above each bar using schematic diagram of inflorescences. The probability of flower retention decreased significantly when basal fruits were present. Differing letters above bars show significant differences based on *post hoc* Tukey’s test for all pairwise comparisons of the inflorescence treatments. The error bars show profile likelihood-based 95 % confidence intervals. *n* = 33, 33 and 18.

The artificial oviposition treatment did not significantly affect flower retention **[see****Supporting Information—Table S1****]**. Hence, we only present the results of the effect of presence of basal fruits on retaining distal flowers. Inflorescences with late-opening distal flowers with already-existing basal fruits retained a significantly lower number of flowers than inflorescences without fruits (*P* = 0.003; Fig. 1) **[see****Supporting Information—Tables S2** and S3**]**. On average, inflorescences with already-existing basal fruits retained less than one out of three distal flowers while inflorescences without already-existing basal fruits retained one to two distal flowers. Further, neither inflorescence treatment nor oviposition level significantly affected the average mass of fruits from experimental flowers retained in the experiment **[see****Supporting Information—Table S4****]**.

We counted the number of infertile white seeds produced by a subset of the fruits collected from the no and high oviposition treatments of the experiment to determine the effectiveness of our oviposition treatment. These fruits produced 351 ± 14 seeds (mean ± SE), of which 38.8 ± 0.1 % (mean ± SE) were infertile white seeds. The number of white seeds did not differ significantly between fruits with no and high artificial oviposition treatments **[see****Supporting Information—Table S5****]**.

### Flower retention and fruit size in natural population

**Figure 2.**
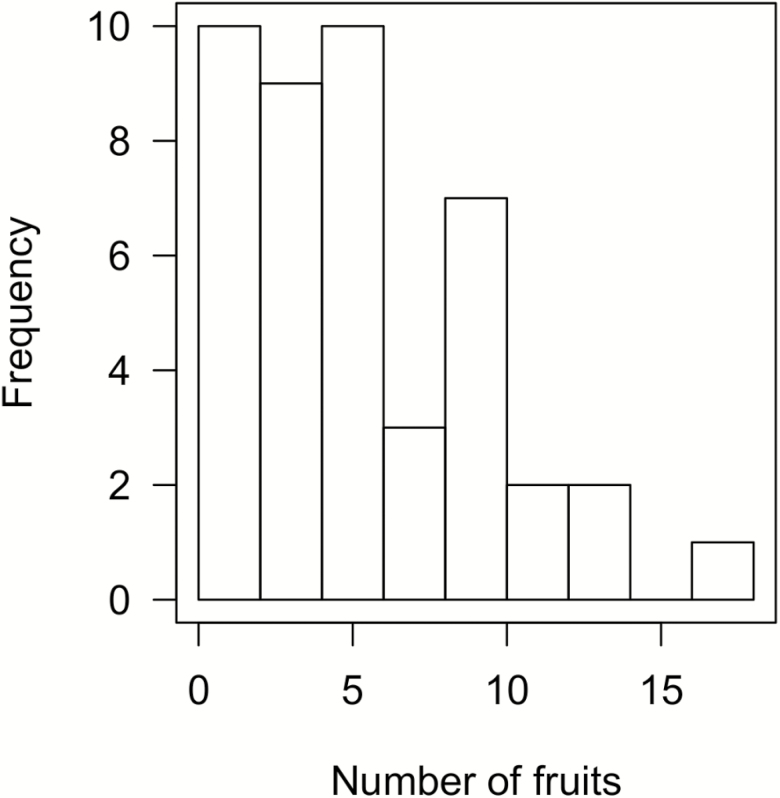
Frequency distribution of number of fruits on naturally pollinated inflorescences with at least one fruit on rosettes with basal diameters within the range of rosettes used in the experiment. *n* = 44 inflorescences.

We used the fruit set distribution in a natural population in the vicinity of the experiments to evaluate if the production of six fruits is close to the upper limit plants of similar size can produce. At the naturally pollinated *Y. glauca* patch, each inflorescence retained 7 ± 0.7 % of its flowers (mean ± SE, *n* = 75 inflorescences), where each fruiting inflorescence produced 6.8 ± 0.7 fruits (mean ± SE) (*n* = 57 inflorescences). *Yucca glauca* rosettes with basal diameters within the range of rosettes used in the experiment produced on average 5.8 ± 0.6 (mean ± SE) fruits with a maximum of 18 fruits in the same year as our experiment (see Fig. 2 for frequency distribution of number of fruits set). Of these, 34 % inflorescences produced more than six fruits, i.e. the maximum fruits that could be produced in our experiment.

Overall, the number of fruits produced varied along naturally pollinated inflorescences. Fruiting inflorescences produced on average significantly more fruits from the middle flowers than the top or bottom flowers, with the bottom flowers producing the least number of fruits (*P* < 0.05) (Table 1). A fruit with a tapering base, as opposed to a rounded base indicates partial fertilization of ovules due to limited pollen. And, increase in locules with constrictions on a fruit indicates increasing level of pollinator oviposition. There was no significant difference in the number of fruits with tapering bases and the number of locules per fruit with constrictions among bottom, middle and top fruits (Table 1).

**Table 1.** The mean ± SE number of fruits, number of fruits with tapering bases (an index of partial fertilization of ovules) and number of locules with constrictions per fruit, from the bottom, middle and top one-third flowers on fruiting inflorescences in the naturally pollinated *Yucca glauca* patch. Numbers in parentheses show number of inflorescences. Number of fruits with tapering bases and number of locules with constrictions were based on fruits collected from 30 inflorescences. Not all inflorescences had fruits in each third of the flowers. Different superscripts within each variable show significantly different means (*P* < 0.05), based on pairwise Wilcoxon rank sum tests with *P*-values adjusted using the Holm–Bonferroni method.

Variables	Bottom	Middle	Top
Number of fruits	1.2 ± 0.2^a^ (57)	3.2 ± 0.4^b^ (57)	2.4 ± 0.4^c^ (57)
Number of fruits with tapering bases	0.4 ± 0.3^e^ (16)	0.4 ± 0.1^e^ (28)	0.3 ± 0.1^e^ (19)
Number of locules with constrictions	1.3 ± 0.4^f^ (16)	1.0 ± 0.2^f^ (28)	1.1 ± 0.3^f^ (19)

**Figure 3.**
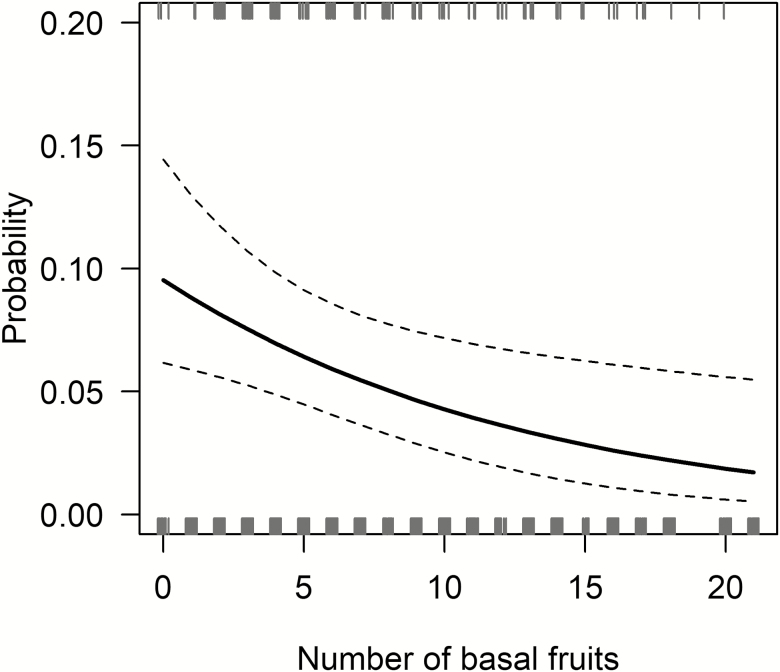
The probability of retaining top flowers decreased with increasing number of basal fruits. Lines are model predicted mean (solid) and 95 % confidence intervals (dashed) at median values of basal diameter of rosettes. Rugs show observed successes (top rugs) and failures (bottom rugs) of retaining top flowers. *n* = 57 inflorescences.

The probability of retaining top flowers significantly decreased with increasing number of basal fruits (*P* = 0.01; Fig. 3) **[see****Supporting Information—Table S6****]**. For inflorescences on rosettes of median basal diameter, in the absence of basal fruits the probability of retaining top flowers was 0.1, which decreased to 0.07 in the presence of five basal fruits. However, there was no significant effect of basal diameter of a rosette on the probability of retaining top flowers (*P* = 0.1) **[see****Supporting Information—Table S6****]**. But, plants with larger basal diameter have inflorescences with more buds and hence more fruits **[see****Supporting Information—Fig. S2A** and B**and****Tables S7** and S8**]**.

Overall, the length of fruits, an index of fruit size, at the naturally pollinated *Y. glauca* patch was 57.61 ± 0.66 mm (mean ± SE). Fruit length interacted significantly with fruit position and whether the fruit was pollen limited, i.e. with a tapering base **[see****Supporting Information—Fig. S3****and****Table S9****]**. Bottom fruits with tapering bases were on average 30 % smaller than bottom fruits without tapering bases. Further, bottom fruits with tapering bases were on average 25 % smaller than middle and top fruits with tapering bases. Sizes of fruits with rounded bases (i.e. without tapering bases) did not significantly differ among fruit positions. We did not find a significant effect of the basal diameter of the rosette and the number of locules with constrictions on fruit size **[see****Supporting Information—Table S9****]**.

## Discussion

### Flower manipulation experiment

#### Resource competition versus architectural hypothesis

We carried out a field experiment to determine whether flower retention in sequentially flowering *Y. glauca* is driven by intrinsic processes related to resource competition between basal fruits and distal flowers and/or architectural effects, and whether these interact with ovule damage due to pollinator oviposition (extrinsic process). We found that, in the absence of basal fruits, the probability of retaining basal and distal flowers was similar, which rejects the hypothesis that architectural effects decrease the probability of retaining distal flowers in *Yucca* spp. Our findings are in line with studies on *Y. glauca* congeners, *Y. filamentosa* (Huth and Pellmyr 1997) and *Y. kanabensis* (Humphries and Addicott 2000, 2004).

We also found that the presence of basal fruits decreased the probability of retaining late-opening distal flowers, which is consistent with the sink strength hypothesis. Preempting a large proportion of resources by existing fruits was unlikely because at the time when distal flowers opened the basal fruits were still in initial stages of development and were only slightly larger than the flowers’ ovaries (S. Jadeja, pers. obs.).

The majority of experimental evidence for the sink strength hypothesis stems from cultivated plants (e.g. Tamas *et al.* 1979; Stephenson 1980; Marcelis *et al.* 2004). These studies were carried out in a controlled laboratory environment with genetically homogenous plants. However, findings from cultivated plants may not necessarily extend to natural plant populations because natural plant populations have larger genetic variation and grow in heterogeneous environments. For example, wild plants are subject to unpredictable disturbances which can lead to large differences between the population dynamics of wild and cultivated plants (Eager *et al.* 2013). Few studies have demonstrated the importance of sink strength on flower retention of wild plants (e.g. Medrano *et al.* 2000; Kliber and Eckert 2004). Hence, our field study on wild *Y. glauca* plants strengthens the empirical support for the role of sink strength of basal fruits in reducing the probability of retaining distal flowers in sequentially flowering plants.

Three hypotheses may explain why plants have evolved mechanisms such as resource sinks that ensure a lower probability of flower retention when basal fruits are already initiated. One is the resource conservation hypothesis, according to which organs that are further in development are preferred by plants for resource investment because they require lower resources in the future to complete development (Nakamura 1986). Conserved resources may be stored for future flowering seasons (Primack and Hall 1990). Another hypothesis is the asset protection principle (Clark 1994). According to this, fruits are more valuable assets to plants than flowers. When fruits are small, they are more vulnerable to risks of damage due to herbivory and storms. Plants can protect their fruits (valuable assets) from such risks by enlarging them quickly to a size that is less vulnerable. Therefore, plants are more likely to allocate resources to enlarge fruits to protect these more valuable assets than retain new flowers that are less valuable assets. A fruiting strategy following the asset protection principle (Clark 1994) may be adaptive for *Yucca* spp. because the risk of losing smaller fruits is high due to herbivory by *Carpophilus* sp. (florivorous beetles) and aphids (Dodd and Linhart 1994; Pellmyr 1995; Huth and Pellmyr 1997), and storms (S. Jadeja, pers. obs.). The third hypothesis is that resource limited plants allocate resources to develop a small number of large fruits in favour of many small fruits because large fruits have larger, more viable seeds (Stanton 1984; Venable 1992; Sakai and Sakai 1995)

#### Effect of artificial oviposition on flower retention

The artificial oviposition treatment did not significantly affect flower retention in our experiment. We evaluated the success of our artificial treatment by comparing the number of damaged ovules in fruits from flowers with and without the artificial oviposition treatment because wounding during ovipositions damages ovules (Marr and Pellmyr 2003). The number of damaged ovules that are visible as infertile white seeds was similar between fruits from experimental flowers with and without the artificial oviposition treatment. This suggests that our artificial oviposition treatment was unsuccessful. However, we cannot completely reject the possibility that the artificial oviposition treatment succeeded, but plants did not abort flowers.

It is possible that the high oviposition treatment (24 ovipositions) was not high enough to elicit a response in our system. Even though we observed female moths to oviposit no more than 30 eggs before leaving a flower (S. Jadeja, pers. obs.), flowers may receive ovipositions from multiple moths. The response to oviposition likely depends on the number of flowers inflorescences produce, which may depend on growing condition and plant genotype. For instance, some *Y. glauca* populations produce inflorescences with up to 1000 flowers (Fuller 1990), which is more than four times the maximum number of flowers observed at our study site. It is possible that plants are more likely to abort flowers with small amount of damage if there are plenty of other flowers to choose from. Also, our experimental flowers were protected from other herbivores. It is possible that the damage caused by 24 artificial ovipositions is extremely small compared to the typical flower damage at our study site.

There is large variation in the number of flowers per inflorescence among congeneric yucca species: *Y. filamentosa* produces 100–475 flowers (Huth and Pellmyr 1997; Marr *et al.* 2000) and *Y. kanabensis* produces 30–150 flowers (Addicott 1998; Shapiro and Addicott 2004). In future experiments it would be interesting to explore the interaction of herbivory level and a species’ potential flower availability in determining abscission responses. These differences in life plant history might contribute to explaining why in earlier experiments 12–18 artificial ovipositions were sufficient to increase flower abortion in yucca plants (*Y. glauca*: Fuller 1990; *Y. filamentosa*: Marr and Pellmyr 2003), while in this study 24 artificial ovipositions were not.

### Flower retention and fruit size in natural population

We quantified the probability of retaining naturally pollinated distal flowers with increasing number of basal fruits. Consistent with experimental results, the probability of retaining top flowers on naturally pollinated inflorescences decreased with increasing number of basal fruits, which also supports the sink strength hypothesis. To our knowledge, the effect of basal fruits on the probability of retaining distal flowers of naturally pollinated *Yucca* spp. has not been studied previously.

In our experiment, the retention probability of basal *Y. glauca* flowers was similar to or higher than distal flowers. However, in naturally pollinated inflorescences basal flowers produced fewer fruits than top flowers, and middle flowers produced the most number of fruits. Higher fruit set from middle fruits is consistent with other studies (Stephenson 1981), including *Y. kanabensis* (Humphries and Addicott 2000, 2004) and *M. cochleare* (Berry and Calvo 1991). One possible reason for the discrepancy between our experimental results and field observations could be high herbivory early in the flowering season in the field, while our experimental flowers were protected from herbivores. For example, a study with a *Y. filamentosa* population showed higher floral herbivory by *Carpophilus* sp. early in the flowering season contributed to low fruit set of early-opening basal flowers (Huth and Pellmyr 1997).

Another likely reason for lower fruit set from early-opening bottom flowers in field populations is poor pollination, while in our experiment we eliminated pollen limitation by hand-pollinating flowers. It is possible that early in the flowering season when bottom flowers open the abundance of nursery pollinators was low. This was observed in *Y. kanabensis* where nursery pollinator *T. altiplanella* visitation peaked in the middle of the flowering season when many inflorescences likely opened their middle flowers (Addicott 1998). Alternatively, bottom flowers may be poorly pollinated due to the nursery pollinator’s preferences for flowers at higher positions on the inflorescence. For example, nursery pollinator *T. altiplanella* prefers ovipositing and pollinating higher flowers (Wilson and Addicott 1998) because flowers higher on the inflorescence are more likely to be receptive and virgin (not visited by other conspecific nursery pollinators).

Fruits with tapering bases indicate partial pollination due to unfertilized ovules from low pollen availability (Humphries and Addicott 2000). We detected that the partial pollination significantly reduced the size of bottom fruits, but we did not detect a similar significant relationship in fruits from middle or top flowers. This suggests that bottom flowers were much more pollen limited than middle and top flowers. This raises the question, why do plants retain partially fertilized early-opening flowers with fewer viable seeds when they could abort those flowers, and retain flowers that open later in the flowering season? Perhaps, if plants abort basal flowers early in the flowering season, their future flowers may not receive pollen, or may be damaged due to herbivory. Therefore, it may not be adaptive for the plants to abort early-opening pollinated flowers, even if they are smaller owing to poor fertilization of ovules.

## Conclusions

Our experimental and observational results from a field population of *Y. glauca* provide support for the hypothesis that strong sink strength of basal fruits reduces the probability of retaining distal flowers but not the architectural effects hypothesis. We discussed three hypotheses that may explain why this strategy has evolved: resource conservation hypothesis, asset protection principle and production of larger more viable seeds.

## Sources of Funding

This research was supported by funds from the J. Ve. Srb Memorial Fund to S.J. B.T. was supported by NSF Grant DEB-655117 and USDA\NIFA Grant 2017-03807.

## Contributions by the Authors

S.J. and B.T. designed the study, developed the methodology, performed the analysis and wrote the manuscript. S.J. collected the data.

## Conflict of Interest

None declared.

## Supporting Information

The following additional information is available in the online version of this article—

**Figure S1.** Experimental set-up for determining flower retention under different inflorescence and oviposition treatments.

**Figure S2.** (A) The number of buds and (B) the number of fruits on inflorescences with increasing basal diameter, an index of rosette size.

**Figure S3.** Length of fruits (bars) in relation to their position along the inflorescence (bottom, middle and top) and shape of the fruit base that indicates whether flowers were pollen limited (tapering base) or not (rounded base).

**Table S1**. Results of the full generalized linear mixed-effects model for the proportion of flowers retained with inflorescence identity as random effect and binomial distribution.

**Table S2.** Results of the final generalized linear mixed-effects model for the proportion of flowers retained with inflorescence identity as random effect and binomial distribution.

**Table S3.** Untransformed mean parameter estimates (Estimate) from Tukey’s all pairwise comparisons of proportion of flowers retained among inflorescence treatments from the final model.

**Table S4.** Results of the full linear model for the average mass of fruits retained on inflorescences with at least one fruit formed from experimental flowers.

**Table S5.** Results of the generalized linear model with quasipoisson distribution to determine effectiveness of the artificial oviposition treatment.

**Table S6.** Results of the generalized linear mixed-effects model with binomial distribution for the probability of retaining top flowers with inflorescence identity as a random effect.

**Table S7.** Results of the generalized linear model with Poisson distribution for the number of buds produced on an inflorescence.

**Table S8.** Results of the generalized linear model with Poisson distribution for the number of fruits matured on an inflorescence.

**Table S9.** Results of the linear mixed-effects model with inflorescence identity as random effect for the length of fruits (mm).

Supporting Information
