# Supplementary material for: Presence of fruits decreases probability of retaining flowers in a sequentially flowering plant
Source: AoB Plants. 2018 May 23;10(3):ply033. doi: 10.1093/aobpla/ply033 (PMC6007677; doi:10.1093/aobpla/ply033)
Supplement: Supporting Information [file ply033_suppl_supporting_information.docx]

## SUPPORTING INFORMATION


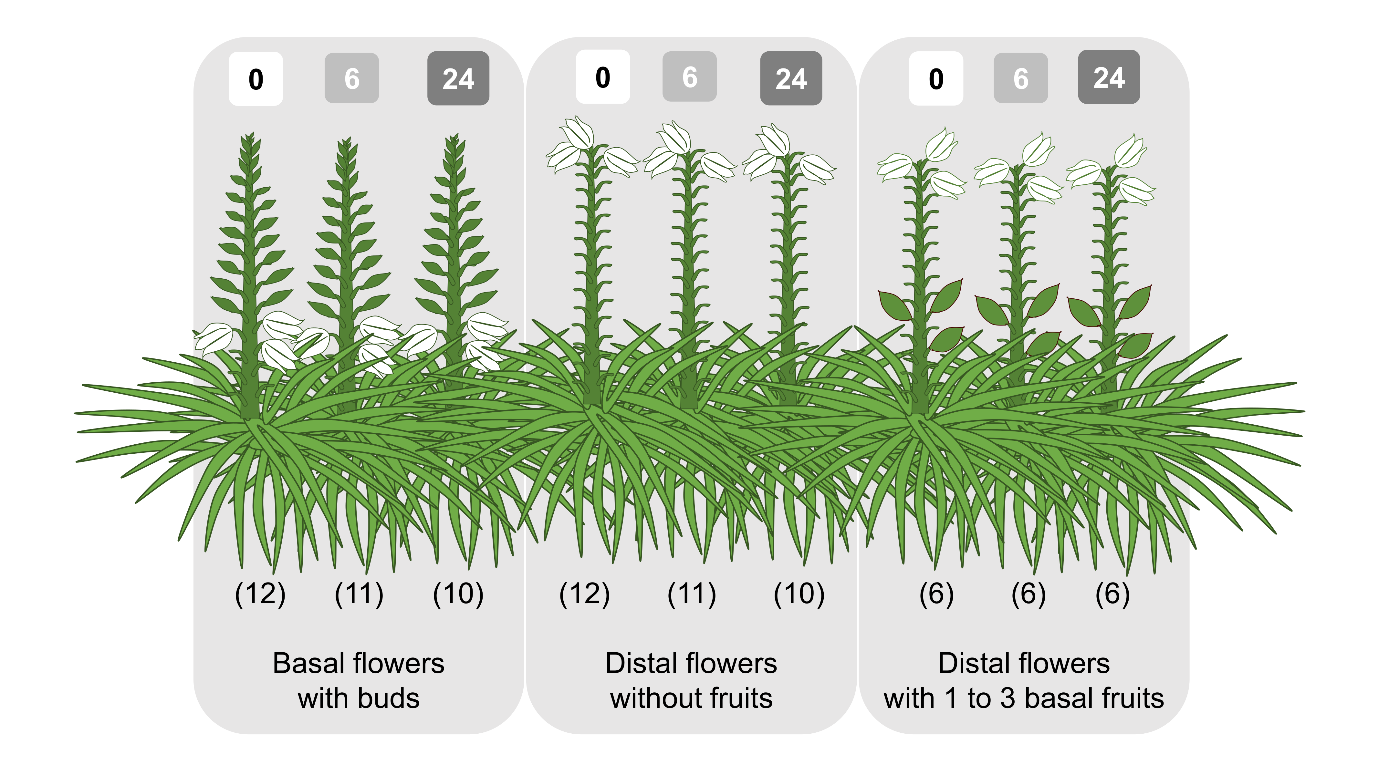


**Figure S1.** Experimental set-up for determining flower retention under different inflorescence and oviposition treatments. Schematic diagram shows *Yucca glauca* inflorescences in each treatment with experimental flowers (white), buds (light green), and basal fruits (dark green). Numbers above inflorescences indicate the number of artificial ovipositions applied to each experimental flower. The numbers in parentheses below inflorescences indicate samples sizes after discarding inflorescences with beetle damage and inflorescences that did not form at least one fruit for the inflorescence treatment with one to three basal fruits.


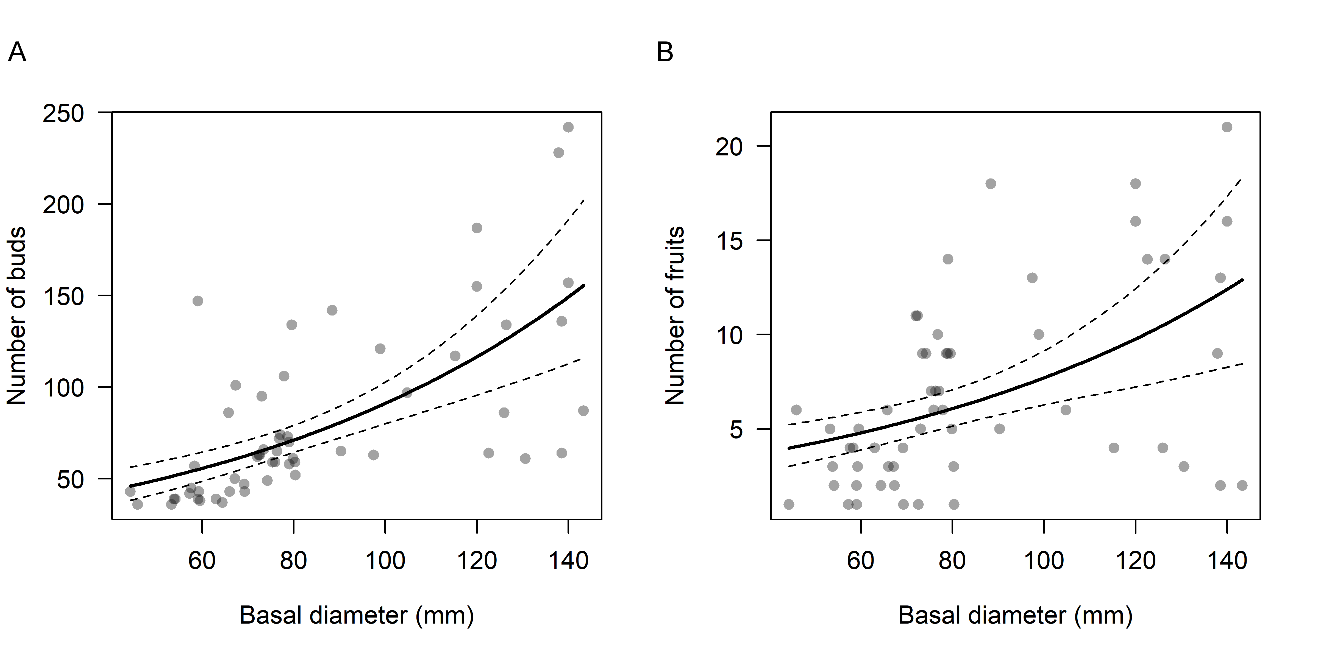


**Figure S2.** (A) The number of buds and (B) the number of fruits on inflorescences with increasing basal diameter, an index of rosette size. Lines are model predicted means (solid) and bootstrapped 95% CIs (dashed). Points are inflorescences, and, overlapping points are indicated by a darker colour. n = 57 inflorescences.


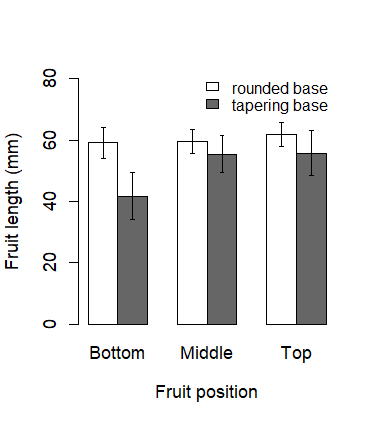


**Figure S3.** Length of fruits (bars) in relation to their position along the inflorescence (bottom, middle and top). The shape of the fruit base indicates whether flowers were pollen limited (tapering base, filled bars) or not (rounded base, open bars). Bars are model predicted means and error bars are 95% CIs. n = 26, 7, 104, 10, 76, and 6 fruits (bars from left to right). Bottom, middle, and top fruits are from 16, 28, and 19 inflorescences. Data are from a total of 30 inflorescences but not all have fruits in each third of the inflorescence. n = 57 inflorescences.

| **Table S1**. Results of the full generalized linear mixed-effects model for the proportion of flowers retained with inflorescence identity as random-effect and binomial distribution. We display the untransformed means of the estimated fixed variables (Estimate) for the effect of inflorescence treatments, artificial oviposition treatments (O) and their interaction on the proportion of flowers retained. SE indicates standard errors, and n is the sample size (number of inflorescences). Variance of the random effect was 1.79. | | | | | |
| --- | --- | --- | --- | --- | --- |
| Parameter | n | Estimate | SE | z value | Pr(>\|z\|) |
| Intercept  (distal flowers - basal fruits**:**high O) | 10 | -0.00 | 0.61 | 0 | 1 |
| basal flowers + buds**:**high O | 10 | -0.98 | 0.89 | -1.11 | 0.27 |
| distal flowers + basal fruits**:**high O | 6 | -1.67 | 1.06 | -1.57 | 0.12 |
| distal flowers – basal fruits**:**low O | 11 | -0.43 | 0.85 | -0.50 | 0.62 |
| distal flowers – basal fruits**:**no O | 12 | 1.12 | 0.84 | 1.32 | 0.19 |
| basal flowers + buds**:**low O | 11 | 2.15 | 1.23 | 1.75 | 0.08 |
| distal flowers + basal fruits**:**low O | 6 | 0.5 | 1.48 | 0.34 | 0.74 |
| basal flowers + buds**:**no O | 12 | -0.14 | 1.19 | -0.11 | 0.91 |
| distal flowers + basal fruits**:**no O | 6 | -1.19 | 1.49 | -0.8 | 0.43 |

| **Table S2.** Results of the final generalized linear mixed-effects model for the proportion of flowers retained with inflorescence identity as random-effect and binomial distribution. We display the untransformed means of the estimated fixed variables (Estimate) for the effect of inflorescence treatment on the proportion of flowers retained. SE indicates standard errors, and n is the sample size (number of inflorescences). Variance of the random effect was 2.11. Significant p-values (Pr<0.05) are in bold. | | | | | |
| --- | --- | --- | --- | --- | --- |
| Parameter | n | Estimate | SE | z value | Pr(>\|z\|) |
| Intercept  (distal flowers – basal fruits) | 33 | 0.27 | 0.36 | 0.74 | 0.46 |
| basal flowers + buds | 33 | -0.29 | 0.5 | -0.58 | 0.56 |
| distal flowers + fruits | 18 | -1.99 | 0.66 | -3.02 | **0.003** |

| **Table S3.** Untransformed mean parameter estimates (Estimate) from Tukey's all pairwise comparisons of proportion of flowers retained among inflorescence treatments from the final model. SE indicates standard errors. Significant p-values (Pr<0.05) are in bold. | | | | |
| --- | --- | --- | --- | --- |
| Pairwise comparisons (using final model) | Estimate | SE | z value | Pr(>\|z\|) |
| basal flowers + buds / distal flowers – basal fruits | -0.29 | 0.50 | -0.58 | 0.83 |
| distal flowers – basal fruits / distal flowers + basal fruits | -1.99 | 0.66 | -3.02 | **0.007** |
| distal flowers + basal fruits / basal flowers + buds | -1.69 | 0.65 | -2.6 | **0.024** |

| **Table S4.** Results of the full linear model for the average mass of fruits retained on inflorescences with at least one fruit formed from experimental flowers. We display means of the parameter estimates (Estimate) for the effect of inflorescence treatment and artificial oviposition treatments (O) and their interaction on the average mass of fruits retained from experimental flowers. SE indicates standard errors, and n is the sample size (number of inflorescences). Significant p-values (Pr<0.05) are in bold. | | | | | |
| --- | --- | --- | --- | --- | --- |
| Parameter | n | Estimate | SE | z value | Pr(>\|z\|) |
| Intercept (distal flowers - basal fruits**:**high O) | 8 | 58.38 | 6.26 | 9.32 | **<0.0001** |
| basal flowers + buds**:**high O | 5 | -6.31 | 10.1 | -0.63 | 0.54 |
| distal flowers + basal fruits**:**high O | 2 | 2.16 | 14.01 | 0.15 | 0.88 |
| distal flowers – basal fruits**:**low O | 7 | -11.49 | 9.17 | -1.25 | 0.22 |
| distal flowers – basal fruits**:**no O | 10 | -8.34 | 8.40 | -0.99 | 0.33 |
| basal flowers + buds**:**low O | 10 | 17.33 | 13.35 | 1.3 | 0.20 |
| distal flowers + basal fruits**:**low O | 4 | -16.41 | 17.87 | -0.92 | 0.36 |
| basal flowers + buds**:**no O | 7 | 17.19 | 13.35 | 1.29 | 0.20 |
| distal flowers + basal fruits**:**no O | 2 | -3.23 | 19.61 | -0.17 | 0.87 |

| **Table S5.** Results of the generalized linear model with quasipoisson distribution to determine effectiveness of the artificial oviposition treatment. We display the untransformed means of the estimated variables (Estimate) for the effect of high and no artificial oviposition treatment on the number of infertile white seeds in experimental fruits. SE indicates standard errors, and n is the sample size (number of fruits). Significant p-values (Pr<0.05) are in bold. | | | | | |
| --- | --- | --- | --- | --- | --- |
| Parameter | n | Estimate | SE | t value | Pr(>\|t\|) |
| Intercept (high oviposition) | 12 | 4.72 | 0.20 | 23.50 | **<0.0001** |
| no oviposition | 10 | 0.28 | 0.28 | 1.00 | 0.33 |

| **Table S6.** Results of the generalized linear mixed effects model with binomial distribution for the probability of retaining top flowers with inflorescence identity as a random effect. We display the untransformed mean of parameter estimates (Estimate) for the effect of the number of basal fruits and basal diameter (mm) of inflorescence's rosette. SE indicates standard errors. n = 1552 flowers across 57 inflorescences. Significant p-values (Pr<0.05) are in bold. Variance of the random effect was 0.87. | | | | |
| --- | --- | --- | --- | --- |
| Parameter | Estimate | SE | z value | Pr(>\|z\|) |
| Intercept | -3.12 | 0.56 | -5.52 | **<0.001** |
| number of basal fruits | -0.09 | 0.03 | -2.48 | **0.01** |
| basal diameter | 0.01 | 0.01 | 1.67 | 0.1 |

| **Table S7.** Results of the generalized linear model with Poisson distribution for the number of buds produced on an inflorescence. We display the untransformed mean of parameter estimates (Estimate) for the effect of the basal diameter (mm) of the inflorescence's rosette. SE indicates standard errors. n = 57 inflorescences. Significant p-values (Pr<0.05) are in bold. | | | | |
| --- | --- | --- | --- | --- |
| Parameter | Estimate | SE | z value | Pr(>\|z\|) |
| Intercept | 3.28 | 0.05 | 68.33 | **<0.001** |
| basal diameter | 0.01 | 0.0005 | 25.66 | **<0.001** |

| **Table S8.** Results of the generalized linear model with Poisson distribution for the number of fruits matured on an inflorescence. We display the untransformed mean of parameter estimates (Estimate) for the effect of the basal diameter (mm) of the inflorescence's rosette. SE indicates standard errors. n = 57 inflorescences. Significant p-values (Pr<0.05) are in bold. | | | | |
| --- | --- | --- | --- | --- |
| Parameter | Estimate | SE | z value | Pr(>\|z\|) |
| Intercept | 0.86 | 0.16 | 5.24 | **<0.001** |
| basal diameter | 0.01 | 0.002 | 7.15 | **<0.001** |

| **Table S9.** Results of the linear mixed-effects model with inflorescence identity as random effect for the length of fruits (mm). We show the means of estimate values (Estimate) for the effect of basal diameter (mm) of inflorescence's rosette, whether the base of the fruit is tapering or rounded, the number of locules with constrictions (cons.), and position of fruit along the inflorescence. SE indicates standard errors and df indicates degrees of freedom. Significant p-values (Pr<0.05) are in bold. Variance of the random effect was 7.18. n = 229 fruits on 30 inflorescences. | | | | | |
| --- | --- | --- | --- | --- | --- |
| Parameter | Estimate | SE | df | t value | Pr(>\|t\|) |
| (Intercept) (bottom fruit:rounded base) | 56.25 | 7.01 | 189 | 8.03 | **<0.001** |
| basal diameter:bottom fruit | 0.03 | 0.06 | 28 | 0.4 | 0.7 |
| tapering base:bottom fruit | -17.46 | 4.06 | 189 | -4.30 | **<0.001** |
| cons.:bottom fruit | -0.22 | 1.04 | 189 | -0.21 | 0.83 |
| middle fruit:rounded base | 9.23 | 6.35 | 189 | 1.45 | 0.15 |
| top fruit:rounded base | 3.08 | 7.58 | 189 | 0.41 | 0.69 |
| basal diameter:middle fruit | -0.08 | 0.06 | 189 | -1.48 | 0.14 |
| basal diameter:top fruit | -0.01 | 0.06 | 189 | -0.12 | 0.91 |
| cons.:middle fruit | 1.08 | 1.15 | 189 | 0.94 | 0.35 |
| cons.:top fruit | 0.56 | 1.19 | 189 | 0.47 | 0.64 |
| tapering base:middle fruit | 13.33 | 4.83 | 189 | 2.76 | **0.01** |
| tapering base:top fruit | 11.38 | 5.18 | 189 | 2.2 | **0.03** |
